# Insights into the phylogeny and chloroplast genome evolution of *Eriocaulon* (Eriocaulaceae)

**DOI:** 10.1186/s12870-023-04034-z

**Published:** 2023-01-14

**Authors:** Enze Li, Kangjia Liu, Rongyan Deng, Yongwei Gao, Xinyu Liu, Wenpan Dong, Zhixiang Zhang

**Affiliations:** Laboratory of Systematic Evolution and Biogeography of Woody Plants, School of Ecology and Nature Conservation, Beijing Forestry University, Beijing, 100083 China

**Keywords:** *Eriocaulon*, Phylogenetic relationships, Chloroplast genome, Divergence time

## Abstract

**Background:**

*Eriocaulon* is a wetland plant genus with important ecological value, and one of the famous taxonomically challenging groups among angiosperms, mainly due to the high intraspecific diversity and low interspecific variation in the morphological characters of species within this genus. In this study, 22 samples representing 15 *Eriocaulon* species from China, were sequenced and combined with published samples of *Eriocaulon* to test the phylogenetic resolution using the complete chloroplast genome. Furthermore, comparative analyses of the chloroplast genomes were performed to investigate the chloroplast genome evolution of *Eriocaulon.*

**Results:**

The 22 *Eriocaulon* chloroplast genomes and the nine published samples were proved highly similar in genome size, gene content, and order. The *Eriocaulon* chloroplast genomes exhibited typical quadripartite structures with lengths from 150,222 bp to 151,584 bp. Comparative analyses revealed that four mutation hotspot regions (*psbK-trnS*, *trnE-trnT*, *ndhF-rpl32*, and *ycf1*) could serve as effective molecular markers for further phylogenetic analyses and species identification of *Eriocaulon* species. Phylogenetic results supported *Eriocaulon* as a monophyletic group. The identified relationships supported the taxonomic treatment of section *Heterochiton* and *Leucantherae*, and the section *Heterochiton* was the first divergent group. Phylogenetic tree supported *Eriocaulon* was divided into five clades. The divergence times indicated that all the sections diverged in the later Miocene and most of the extant *Eriocaulon* species diverged in the Quaternary. The phylogeny and divergence times supported rapid radiation occurred in the evolution history of *Eriocaulon*.

**Conclusion:**

Our study mostly supported the taxonomic treatment at the section level for *Eriocaulon* species in China and demonstrated the power of phylogenetic resolution using whole chloroplast genome sequences. Comparative analyses of the *Eriocaulon* chloroplast genome developed molecular markers that can help us better identify and understand the evolutionary history of *Eriocaulon* species in the future.

**Supplementary Information:**

The online version contains supplementary material available at 10.1186/s12870-023-04034-z.

## Background

The family Eriocaulaceae includes 11 genera and about 1,400 species that occur primarily in the neotropics [1, 2]. Molecular phylogenetic studies showed that Eriocaulaceae is divided into two subfamilies (Eriocauloideae and Paepalanthoideae) [1]. The Eriocauloideae includes two genera, *Eriocaulon* L. and *Mesanthemum* Körn. *Mesanthemum* is only distributed in Africa, and most *Eriocaulon* species are confined to tropical and subtropical regions. There are three centers of species diversity, namely Africa (contains 111 species), the Americas (contains 122 species), and Asia (contains 220 species) [3, 4].

*Eriocaulon,* which includes about 470 species, is characterized by diplostemonous flowers with twice as many stamens as petals, nectar glands on the apices of the petals, and staminate and pistillate flowers with free petals [1, 5, 6]. These nectaries produce fluid that attracts insects, indicating insect pollination [5]. The species of the genus are mainly perennial herbs that grow in moist habitats or shallow wetlands. As a species-rich and widely distribution genus of wetland plants, *Eriocaulon* plays a significant role in the ecosystem [7].

The taxonomy of *Eriocaulon* is very difficult due to the high intraspecific diversity and low interspecific variation in the morphological characters within the genus [2, 8–11]. Hooker referred to the *Eriocaulon* as “the most difficult of classification, presenting no good sectional characters.” Several systematic studies have focused on *Eriocaulon* in Australia [9, 10] and India [12] using the molecular and morphological evidence. In the last ten years, many new *Eriocaulon* species have been described in India [13–17], Southeast Asia [18–21], and Brazil [22–24].

According to the Flora of China, there are 35 species in China [8]. Two subgenera (*Trimeranthus* Nakai and *Eriocaulon* Nakai) were classified of the Chinese species [25] Subgen. *Trimeranthus* was further divided into three sections: sect. *Spathopeplus* Nakai, sect. *Leucocephala* Nakai, and sect. *Macrocaulon* Ruhl.. Based on the morphological characteristics of the seeds and flowers, we established an infrageneric system [3]. The infrageneric classification recognized two subgenera and 10 sections. Phylogenetic analyses of the Eriocaulaceae strongly supported the monophyly of *Eriocaulon* [1, 2]. Only a few molecular studies sought to resolve phylogenetic relationships at the species level within this widespread genus. Davies et al. [9] resolved the taxonomy of the *E. carsonii* complex in Australia with amplified fragment length polymorphism (AFLP) genetic markers. Recently, Larridon et al. [4] used five markers, including four chloroplast markers and one nuclear marker, to create the first molecular phylogenetic study for the genus. Darshetkar et al. [12] focused on the Indian *Eriocaulon* species that 552 accessions from 66 *Eriocaulon* species were analyzed. This phylogenetic study used ITS and *trnL–F*, yielding three major clades of Indian *Eriocaulon* species. However, the phylogenetic relationships of Chinese *Eriocaulon* species are poorly understood. The markers used in the *Eriocaulon* phylogeny offer less information and the phylogenetic relationships are poorly resolved. More genetic markers are needed to access the phylogenetic relationships of *Eriocaulon* species in China.

The chloroplast genome is smaller than the plant mitochondrial and nuclear genomes, and the chloroplasts play a crucial role in photosynthesis [26, 27]. The chloroplast genome exhibits a conserved quadripartite structure of a large single-copy (LSC), a small single-copy (SSC), and two inverted repeat regions (IRs). Most angiosperms exhibit maternal inheritance [28, 29], and the chloroplast genomes are structurally stable during evolution, with mutation rates that are between those shown in the mitochondrial and nuclear genomes [30]. Therefore, the chloroplast genome provides an ideal model for genomic evolution and molecular markers for resolving phylogenetic relationships [31–33]. The chloroplast sequences were the first to be used in molecular evolution [34], and considerable attention has been paid to the evolutionary rate variations among genes or lineages in the chloroplast genome [31].

Recently, more and more studies have shown that variation in chloroplast genomes provides effective information that can be used to resolve phylogenetic relationships at multiple taxonomy levels, especially in taxonomically complex groups [35, 36]. For example, chloroplast genome data had resolved the systematic positions of enigmatic taxa in Saxifragales [37] and shed lights on the intergeneric relationships and spatio-temporal evolutionary history of *Melocanninae* (Poaceae) [36]. Moreover, the chloroplast genome sequences showed variations at the intraspecies levels, and revealed the genetic difference and diversity of endangered species [38, 39] and cultivated species [40, 41].

In this study, we assembled the whole chloroplast genomes of 22 *Eriocaulon* samples and combined them with nine published samples in GenBank. These samples included half of the species in China, and the taxonomic status of some species were unresolved. Furthermore, we analyzed most of the chloroplast gene sequences in GenBank. Our specific goals were as follows: (a) to compare the chloroplast genome structures within the genus *Eriocaulon*; (b) to identify the mutation hotspot regions as potential chloroplast markers for species identification and phylogeny; (c) to infer and test the phylogenetic relationships and divergence time among the *Eriocaulon* species in China using the whole chloroplast genome; (d) to include the chloroplast gene sequences from GenBank to infer the deep relationships of *Eriocaulon* species in the world.

## Results

### General features of *Eriocaulon* chloroplast genomes

**Fig. 1:**
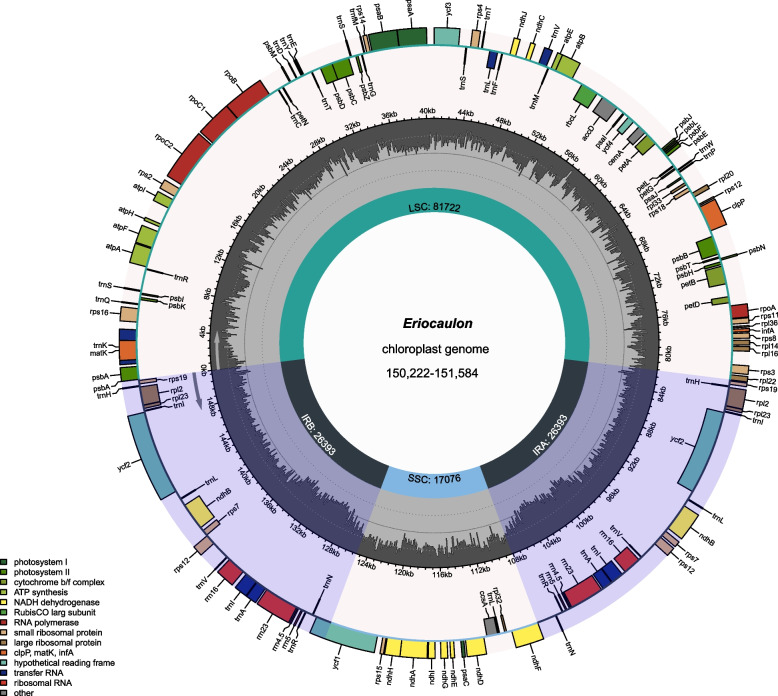
Structural and gene map of the *Eriocaulon* chloroplast genome

The length of the 31 chloroplast genomes varied from 150,222 bp (*E.* sp. 02) to 151,584 bp (*E. australe* 01) (Table 1 and Table S1). The *Eriocaulon* chloroplast genome exhibited typical quadripartite structures (Fig. 1). The IR regions (ranging from 25,950 bp (*E. schochianum*) to 26,532 bp (*E. decemflorum* 01)) were separated by an LSC region ranging from 80,367 bp (*E. oryzetorum*) to 81,722 bp (*E. australe* 01) and an SSC region ranging from 16,890 (*E. decemflorum* 02) to 17,104 bp (*E. australe* 03). The GC content in the *Eriocaulon* chloroplast genomes was 35.7–35.9% (Table 1). There were 113 unique genes in the chloroplast genome of *Eriocaulon* species, including 79 protein-coding genes, 30 tRNA genes, and four rRNA genes. Among the protein-coding genes, 44 genes were associated with photosynthesis, and 25 were related to self-replication.

**Table 1:** Summary of the chloroplast genomes for 31 *Eriocaulon* samples

Section	Species	Total length (bp)	LSC (bp)	IR (bp)	SSC (bp)	Total GC content (%)	Total genes	Protein coding genes	rRNA genes	tRNA genes
Apoda	*E. alpestre* 01	151,372	81,354	26,513	16,992	35.8	114	80	4	30
Apoda	*E. alpestre* 02	151,398	81,340	26,514	17,030	35.8	114	80	4	30
Apoda	*E. alpestre* 03	151,343	81,361	26,483	17,016	35.8	114	80	4	30
Heterochiton	*E. australe* 01	151,584	81,722	26,393	17,076	35.7	114	80	4	30
Heterochiton	*E. australe* 02	150,770	81,403	26,132	17,103	35.7	114	80	4	30
Heterochiton	*E. australe* 03	151,498	81,592	26,401	17,104	35.8	114	80	4	30
Simplices	*E. brownianum*	151,432	81,533	26,457	16,985	35.8	114	80	4	30
Apoda	*E. buergerianum* 01	151,383	81,331	26,507	17,038	35.8	114	80	4	30
Apoda	*E. buergerianum* 02	151,366	81,334	26,507	17,018	35.8	114	80	4	30
Apoda	*E. buergerianum* 03	151,389	81,347	26,507	17,028	35.8	114	80	4	30
Leucantherae	*E. cinereum* 01	150,725	80,741	26,523	16,938	35.8	114	80	4	30
Leucantherae	*E. cinereum* 02	150,708	80,733	26,523	16,929	35.9	114	80	4	30
Nasmythia	*E. decemflorum* 01	151,408	81,424	26,532	16,920	35.9	114	80	4	30
Nasmythia	*E. decemflorum* 02	151,032	81,152	26,495	16,890	35.9	114	80	4	30
Apoda	*E. faberi* 01	151,361	81,329	26,507	17,018	35.8	114	80	4	30
Apoda	*E. faberi* 02	151,361	81,329	26,507	17,018	35.8	114	80	4	30
-	*E. fistulosum*	151,415	81,493	26,460	17,002	35.9	114	80	4	30
Anisopetalae	*E. henryanum* 01	151,051	81,171	26,451	16,978	35.8	114	80	4	30
Anisopetalae	*E. henryanum* 02	151,010	81,119	26,456	16,979	35.8	114	80	4	30
Apoda	*E. miquelianum*	151,344	81,304	26,507	17,026	35.8	114	80	4	30
Simplices	*E. nantoense*	151,389	81,509	26,442	16,996	35.8	114	80	4	30
Simplices	*E. nepalense* 01	150,947	81,064	26,451	16,981	35.8	114	80	4	30
Simplices	*E. nepalense* 02	150,973	81,090	26,451	16,981	35.9	114	80	4	30
Simplices	*E. oryzetorum*	150,255	80,367	26,441	17,006	35.8	114	80	4	30
Simplices	*E. schochianum*	150,450	81,570	25,950	16,980	35.8	114	80	4	30
Simplices	*E.* sp. 01	151,462	81,579	26,449	16,985	35.8	114	80	4	30
Leucantherae	*E.* sp. 02	150,222	80,611	26,349	16,913	35.9	114	80	4	30
Leucantherae	*E. tokinense* 01	150,951	81,185	26,411	16,944	35.7	114	80	4	30
Leucantherae	*E. tokinense* 02	150,900	81,146	26,411	16,932	35.7	114	80	4	30
Disepala	*E. truncatum* 01	151,052	81,117	26,452	17,031	35.9	114	80	4	30
Disepala	*E. truncatum* 02	151,057	81,122	26,452	17,031	35.9	114	80	4	30

The boundaries between IR and SC regions were compared in the 18 *Eriocaulon* species (Fig. 1). The *Eriocaulon* SC/IR junctions were highly conserved. the LSC/IRb junction was located in *rpl22*, while the IRb/SSC junction was located in the *ndhF*, and IRb expanded progressively from the IR regions to *ndhF*. The IRa/SSC junction was found within the *ycf1* and the IRa/LSC border was adjacent to the *psbA*.

For all *Eriocaulon* species, 64 types of codons encoding 20 amino acids were detected (Figure S1). The total number of codons was 22,336–22,571. AUU was the most-used codon (982–1,000 instances), whereas CGG was the least (65–71 instances). The RSCU values are shown in Figure S1, and the values for all codons ranged from 0.26 to 2.27 in the *Eriocaulon* chloroplast genome. The RSCU values of 30 codons were greater than 1.00 in all *Eriocaulon* chloroplast genomes and all of them ended with A/U, except for UUG.

### SSR polymorphisms and long repeat structure

We total identified 777 SSRs in the 18 *Eriocaulon* chloroplast genomes (Table 2). The number of SSRs in *Eriocaulon* ranged from 33 to 58, with an average of 43. Dinucleotide repeats were the most common (37.07%), followed by mononucleotide repeats (22.13%), tetranucleotide repeats (21.75%), and trinucleotide repeats (14.41%); pentanucleotide and hexanucleotide repeats were the least common (2.32%). Most of the SSRs were located in the intergenic region of the LSC.

**Table 2:** Numbers and types of SSRs in the 18 *Eriocaulon* chloroplast genomes

**Species**	mono-	di-	tri-	tetra-	penta-	hexa-	Total
*E. alpestre*	11	18	3	12	0	2	46
*E. australe*	16	21	10	10	1	0	58
*E. brownianum*	8	18	8	10	2	1	47
*E. buergerianum*	12	16	4	12	0	2	46
*E. cinereum*	9	15	4	7	0	0	35
*E. decemflorum*	10	14	5	8	1	1	39
*E. faberi*	13	17	4	12	0	2	48
*E. fistulosum*	8	10	5	8	2	0	33
*E. henryanum*	8	18	7	10	0	1	44
*E. miquelianum*	12	15	6	11	0	2	46
*E. nantoense*	8	19	7	8	1	1	44
*E. nepalense*	8	17	7	10	2		44
*E. oryzetorum*	8	14	4	4	2	1	33
*E. schochianum*	7	18	8	10	3	1	47
*E.* sp. 01	7	18	8	11	2	2	48
*E.* sp. 02	9	16	5	7	0	0	37
*E. tokinense*	7	12	10	14	0	1	44
*E. truncatum*	11	12	7	5	2	1	38

**Fig. 2:**
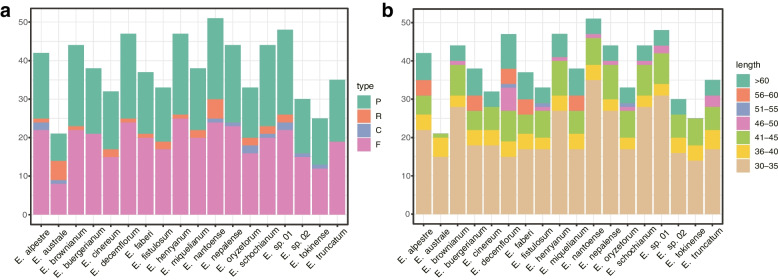
Long-repeat sequences in the *Eriocaulon* chloroplast genomes. **a** Total number of the four repeat types. **b** Number of repeats by length

Four categories of long repeats—forward, reverse, complement, and palindromic—were detected (Fig. 2). There were 8–25 forward repeats, 0–2 reverse repeats, 0–5 complement repeats, and 7—22 palindromic repeats. *E. australe* had the lowest (21) and *E. nantoense* had the highest (51) number of repeats. The repeat sizes ranged from 30 to 86 bp. More than half of the repeats were 30–35 bp long, while only three repeats were 51–55 bp long.

### *Eriocaulon* chloroplast genome variation

The mVISTA results showed the *Eriocaulon* chloroplast genome had collineation, no rearrangement, and high sequence similarity (Figure S2). The *Eriocaulon* chloroplast genomes aligned with a length of 159,226 bp, including 16,502 variable sites (10.36%), and 14,365 parsimony-informative sites (9.02%). The overall nucleotide diversity (π) was 0.02448 (Table 3). The SSC regions had the highest variation and the IR had the lowest sequence divergence. The mean interspecies and intraspecies genetic distances were 0.0279 and 0.0012, respectively. *Eriocaulon* sp. 01 and *E. brownianum* had the lowest genetic distance value (0.003) and *E. australe* and *E. decemflorum* had the highest (0.0431). *Eriocaulon australe* had the highest intraspecies genetic distance (0.0048) among the three samples.

**Table 3:** Sequence divergence of the *Eriocaulon* chloroplast genomes

Regions	Aligned length (bp)	Variable sites		Information sites		Nucleotide Diversity	Number of Haplotypes
		Numbers	%	Numbers	%		
LSC	87,335	11,501	13.17%	10,006	11.46%	0.03131	30
SSC	17,756	2946	16.59%	2580	14.53%	0.04268	30
IR	27,053	1030	3.81%	892	3.30%	0.00848	28
Whole chloroplast genome	159,226	16,502	10.36%	14,365	9.02%	0.02448	30

**Fig. 3:**
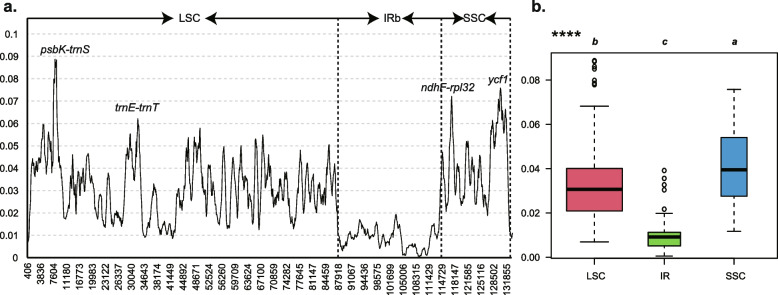
The nucleotide diversity (π) values in the *Eriocaulon* chloroplast genome. Window size: 800 bp, step size: 100 bp. **a** The π values of the windows. **b** Boxplots of π-value differences among the LSC, IR, and SSC regions

Mutation hotspots in the *Eriocaulon* chloroplast genome was identified using the slide window method, and the results are presented in Fig. 3a. The π values ranged from 0 to 0.08872 within an 800-bp window. The π values > 0.06 was defined the mutation hotspots regions. Four peaks were identified, including three noncoding regions (*psbK-trnS*, *trnE-trnT*, and *ndhF-rpl32*) and one coding region (*ycf1*). Two regions (*psbK-trnS* and *trnE-trnT*) were located in the LSC region and the other two (*ndhF-rpl32* and *ycf1*) in the SSC region. The *psbK-trnS* region exhibited the highest π value. This result also showed that the SSC regions had the highest variation and the IR had the lowest sequence divergence (Fig. 3b).

### Molecular evolution of the *Eriocaulon* chloroplast genomes

**Fig. 4:**
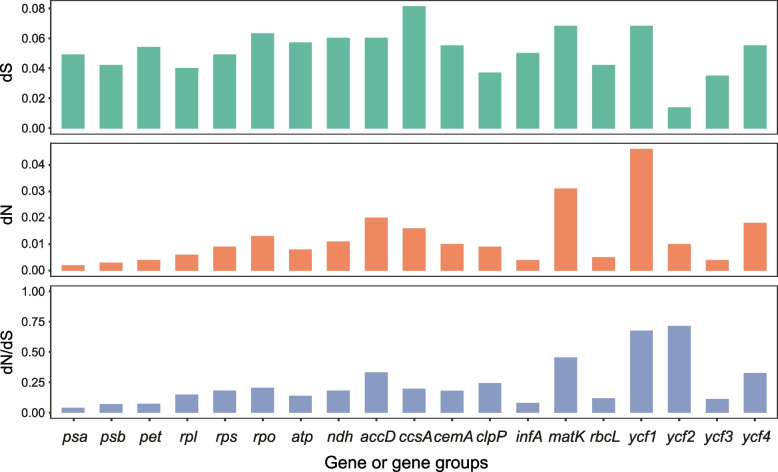
The evolutionary values of dN, dS and ratio (ω) in *Eriocaulon*

The dS, dN, and ω values for the 79 protein-coding genes are shown in Supplemental Table S2. The highest dN value was 0.046 in the *ycf1* gene, and the highest dS value was 0.105 in the *rps15* gene. All the ω values were less than 0.5, indicating the genes were under purifying selection. The t test showed the values of dS, dN, and ω in the genes had significant differences, indicating variable molecular evolution rate among the genes. Among the gene groups, the *rps* group had the highest ω values and the *psa* group had the lowest (Fig. 4). The t test supported the difference of mutation rates among the gene groups.

### Phylogenetic relationships of *Eriocaulon*

The whole chloroplast genome dataset contained 31 *Eriocaulon* chloroplast genome samples and one outgroup of *Paepalanthus alpinus*, among which, 164,361 bp were aligned nucleotide sites, including 27,016 variable sites. The 83-gene dataset contained 73,559 nucleotide sites, including 9,654 variable sites and 5,133 parsimony-informative sites. The phylogenetic relationships of *Eriocaulon* based on the two datasets showed similar topologies (Figure S3). All *Eriocaulon* species formed a monophyletic group (BS = 100/PP = 1) and all relationships among the major clades were strongly supported. All samples of the same species also formed a clade.

The section *Heterochiton* was the first divergent group of *Eriocaulon* and was sister to the remaining species. The section *Leucantherae*, including three species (*E. cinereum*, *E.* sp. 02, and *E. tokinense*), was the second divergent group and was strongly supported. The section *Simplices* (including *E. henryanum*, which belongs to section *Anisopetalae*) was a sister to *Disepala.* The section *Apoda* formed a monophyletic group with high support values (BS = 100/PP = 1) and was a sister to the section *Nasmythia*. The phylogenetic position of *E. fistulosum*, from Australia, was uncertain due to the lower support values (BS = 42/PP = 0.8 in the whole chloroplast genome dataset and BS = 72/PP = 0.99 in the 83-gene dataset). The branch lengths of sections *Apoda* and *Simplices* were very short, indicating that these groups may have undergone rapid radiation.

**Fig. 5:**
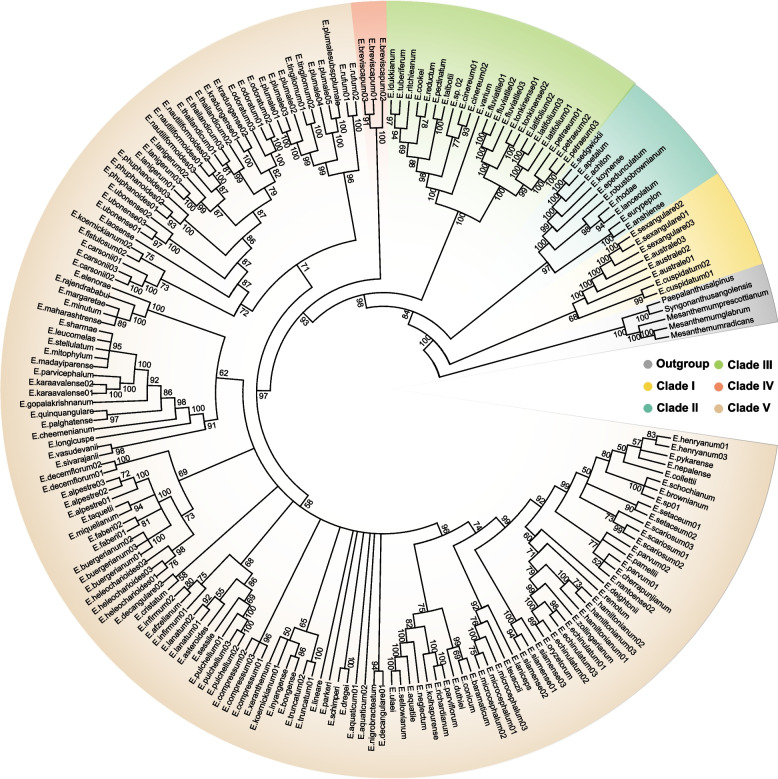
Phylogenetic trees of *Eriocaulon* using the five chloroplast genes

The chloroplast gene dataset contained 197 samples and 121 species of *Eriocaulon* (Table S3)*.* The dataset of five genes included 5,322 aligned sites of which 917 were variable sites. Five clades were supported by the ML tree in *Eriocaulon* (Fig. 5). The Clade I contained the species from the section *Heterochiton*, which was the first divergent group and was sister to the remaining clades. Clade II included ten species which were mainly distributed in India. Clade III consisted of the species of section *Leucantherae*. Clade IV was the singleton, containing *E. breviscapum*. The major *Eriocaulon* species were in the clade V and the subclades in this clade were less well supported.

### Divergence time estimate

**Fig. 6:**
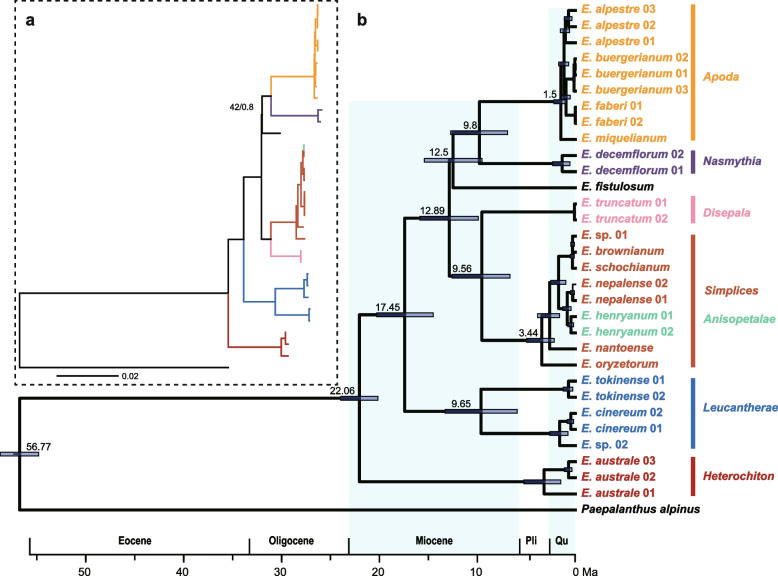
Phylogeny and divergence times of *Eriocaulon.* **a** the tree topology of *Eriocaulon* using the whole chloroplast genome dataset. The number above the lines indicates the ML bootstrap values (BS) and BI posterior probability (PP). BS = 100 and PP = 1.0 are not shown. **b** divergence times of *Eriocaulon*. The blue bars correspond to the 95% highest posterior density (HPD)

Using the 83-gene datasets, the divergence time suggests that the stem and crown ages of *Eriocaulon* were 56.77 Ma (95% highest posterior densities (HPD): 55.88–62.91 Ma) in the early Eocene and 22.06 Ma (95% HPD) during the later Oligocene (Fig. 6). The stem and crown ages of section *Leucantherae* were 17.45 Ma and 9.65 Ma. The split between the section *Anisopetalae* and section *Disepala* occurred at 9.56 Ma, during the later Miocene. The split between the section *Apoda* and *Nasmythia* occurred at 9.8 Ma.

Using the five chloroplast gene dataset, the crown age of *Eriocaulon* was 22.3 Ma, the five clades were divergent from 17.01 Ma to 21.24 Ma showing rapid radiation. Most of species was divergent less than 10 M, starting at the later Miocene (Figure S4). These results indicated that all of the sections or clades had diverged in the later Miocene and most of the extant *Eriocaulon* species diverged in the Quaternary.

## Discussion

### Chloroplast genome evolution of *Eriocaulon*

This study is the first to attempt a comparative analysis of *Eriocaulon* chloroplast genomes. The 31 *Eriocaulon* chloroplast genomes were very similar in overall structure, gene numbers, content and order. However, the length of the chloroplast genome showed noticeable differences compared with other lineages within the genus [32, 42, 43]. The *Eriocaulon* chloroplast genome size ranged from 150,222 bp to 151,584 bp, while the LSC region ranged from 80,367 bp to 81,722 bp (Table 1). The length differences occurred mainly in the LSC regions, while the coding region showed less variation. This suggested that the chloroplast genome size variation of *Eriocaulon* species mainly occurred in the non-coding regions within the LSC region.

Sequences with higher GC content are more stable and have lower mutation rates. Among angiosperms, the overall GC content typically accounts for 30–40% of the chloroplast genome, and the IR region exhibits higher GC content than the LSC and SSC regions [40, 44, 45]. The overall GC content in the *Eriocaulon* chloroplast genomes was 35.7–35.9% and the rRNA genes in the IR regions had a high level of GC content (55.2%), which contributed to the high GC content in the IR region overall (43.2%) compared with that of the LSC region (32.7%) and SSC region (27.8%).

Long sequence repeats in the genomes contribute to genome rearrangement [46–48]. In the *Eriocaulon* chloroplast genomes, 21 (*E. australe*) to 51 (*E. nantoense*) repeats were found in each species. Four types of sequence repeat occur; in previous studies, forward repeats were the most abundant in the chloroplast genome. However, we found almost as many palindromic repeats as forward repeats in the studied species (Fig. 2). SSRs are very abundant in the chloroplast genome and most of them are universal at the interspecies level within the genus or even the family [49, 50]. In the *Eriocaulon* chloroplast genomes, we found 33 to 58 SSR loci. Other studies have shown that the most abundant SSRs were A/T-rich mononucleotide repeats, which was consistent with the chloroplast genome’s common polyA or polyT repeats and rare G or C repeats [35, 51–53]. Dinucleotide repeats were the most common type in the *Eriocaulon* chloroplast genomes (Table 2) and had high AT content.

### Chloroplast markers for *Eriocaulon*

As a famously difficult taxonomic group, effective molecular markers are necessary to rapidly assess genetic divergence and identify species. However, universal or common molecular markers are ineffective for this group [4, 12]. The mutation events are not random and are concentrated in hotspot regions in the chloroplast genome sequences, so variable markers or species barcodes can be identified in the chloroplast genome [32, 54]. Based on the nucleotide diversity analyses, we proposed four regions with high π values with high potential as markers to resolve taxonomic issues in *Eriocaulon* and function as DNA barcodes for species identification.

The intergenic region *psbK-trnS* possess the highest π values (Fig. 3), however, this marker is little used in plant phylogeny. The intergenic region *trnE-trnT* is about 800 bp long and is used in *Camassia* (Agavaceae) [55], *Chamaecrista* sect. *Xerocalyx* [56], and the family Solanaceae [57]. However, this space often contains large A/T-rich regions that may lead to low sequence quality in some groups [58]. In the *Eriocaulon*, we detected an SSR structure (repeat type: AT) within some species. The *ndhF-rpl32*, located in the SSC region with an alignment length of 1,496 bp, has a long history of use in specie identification and plant phylogenetic studies [59]. This region has previously displayed a high level of genetic divergence and is probably the most variable marker at low taxonomic level. The two regions in the coding gene *ycf1* (*ycf1a* and *ycf1b*) are the most variable markers in several plant lineages and are more variable than *matK* and *rbcL* combination [32, 60]. Recently, *ycf1* has been used as the core DNA barcode in the study of plant phylogeny [61–63]. Based on our study, these four divergent markers may be helpful for further phylogenetic and species identification of *Eriocaulon* species.

### Phylogenetics and divergence time of *Eriocaulon*

The relationships derived by using two chloroplast genome datasets were consistent. The phylogenetic resolution of *Eriocaulon* species has been greatly improved in comparison with recently published results [4, 12], with most nodes having 100% support values (Figure S3). However, the five chloroplast genes had the lower resolution and supports *Eriocaulon* species was divided into five clades (Fig. 5). Molecular phylogeny partly supported the taxonomic classification at the section level for the Chinese species in our previous study based on their morphological characteristics (Fig. 5) [3], such as the seed surfaces and calyces of female flowers.

Ma [25] classified the 28 Chinese species of *Eriocaulon* into the two subgenera *Trimeranthus* and *Eriocaulon *sensu (monotypic: *E. decemflorum* Maxim.), according to their flower numbers. We recognized two subgenera of East Asian species [3]. The subgenus *Spathopeplus* Koern, which included seven sections (*Macrocaulon*, *Simplices*, *Anisopetalae*, *Heterochiton*, *Disepala*, *Leucantherae*, and *Nasmythia*), has the sepals of the female flowers fused to some extent into a spathe. The subgenus *Trimeranthus* Nakai, which included three sections (*Macropoda*, *Apoda*, and *Nudicuspa*), has free female sepals. Molecular phylogenetic relationships did not support both taxonomic treatments of the subgenera (Fig. 6 and Figure S3) and not all of the subgenera were monophyletic groups. The section *Heterochiton* included three species in East Asia (clade I in Fig. 5), large herbs that grow 20–60 cm high. This section was the first divergent group in *Eriocaulon* (Fig. 5) [4]. The sections *Simplices* and *Anisopetalae* formed a clade that was supported by their morphological characteristics (Fig. 6 and Figure S3), such as three female sepals with a reduction of the median sepal. There are many more species in section *Simplices* and it is difficult to distinguish them using morphology, as in the *E. nepalense* complex (comprising *E. nepalense*, *E. huzulaefolium*, and *E. nantoense*). *Eriocaulon decemflorum* (section *Nasmythia*) was retrieved as a single-species lineage (Figure S3). This result supports its position as the only member of section *Nasmythia* based on its reduced, dimerous flowers and seed ornamentation structure. The subclades of the Clade V were poorly resolved using the five chloroplast genes (Fig. 5). Larridon et al. [4] divided the Clade V into approximately seven branches, however, owing to the lower supported values, these results were not solid and adding more molecular data is essential for phylogeny of this famous taxonomically challenging group.

Phylogenetic and divergence time analysis indicated that the *Eriocaulon* species may have undergone rapid radiation. The divergence time analysis results indicated that *Eriocaulon* originated in the early Eocene (Fig. 6). There were two significant periods of rapid diversification of *Eriocaulon*. The first was in the early Miocene, which led to the major lineages of the extant *Eriocaulon* species. During this period, due to the higher temperatures [64–67], suitable habitats for *Eriocaulon* were fragmented through aridification, which led to the first rapid radiation. The second period was in the Quaternary, which led to most of the extant *Eriocaulon* species. After 5 Ma, the global temperature decreased sharply after a short period of global warming [68], providing a diverse range of habitats and further increasing the species diversity of *Eriocaulon.*

## Conclusions

In this work, we sequenced and assembled the complete chloroplast genome sequences of 22 samples representing 15 *Eriocaulon* species. By adding published samples of *Eriocaulon*, comparative genomics indicated that the *Eriocaulon* chloroplast genomes were relatively conserved and four mutation hotspot regions emerged as potential variable molecular markers for inferring phylogenetic relationships and species identification. Phylogenetic analysis based on the chloroplast genome supported part of the results of our previous taxonomic treatment study at the section level using morphological characteristics. The world *Eriocaulon* species were divided into five clades and underwent the rapid radiation. Divergence time analysis revealed that *Eriocaulon* originated in the early Eocene and diversified in the later Miocene. Overall, this study demonstrated that the whole chloroplast genome sequences displayed variable information to resolve phylogenetic relationships in this difficult-to-characterize genus.

## Methods

### Sample collection and sequencing

We collected 22 samples representing 15 species in China. The sample details are shown in Table S1 and the voucher specimens were deposited at the Museum of Beijing Forestry University. Zhixiang Zhang identified all samples. We also downloaded all of the published complete chloroplast genomes of *Eriocaulon* from GenBank. In total, we obtained 31 samples representing 18 *Eriocaulon* species (Table S1).

Fresh leaves dried in silica gel for DNA extraction. The total genomic DNA was extracted with the mCTAB method [69]. NanoDrop 2000 microspectrophotometer was used to quantify the DNA concentration and quality. Genomic DNA was fragmented randomly into 350 bp segments with an ultrasonicator. A paired-end library was constructed with an insert size of 350 bp and sequenced with the Illumina Hiseq Xten sequencing system at Novegene Co. Ltd. in Tianjin. Approximately 5.0 Gb of raw data were generated for each sample.

### Chloroplast genome assembly and annotation

To obtain high-quality clean reads, Trimmomatic v0.36 [70] was run to cut and remove the adaptors and low-quality reads. GetOrganelle [71] was used to assemble the chloroplast genome and the k-mer length was set to 95. Clean reads were mapped to the assembled chloroplast genome using Geneious Prime (Biomatters Ltd., Auckland, New Zealand) to validate the sequence errors. The complete chloroplast genome was annotated using the perl script Plann [72] with the *Eriocaulon henryanum* (OK539718) as the reference. The errors in the start and stop codon positions of the protein genes were manually checked and adjusted using Geneious Prime [73].

Chloroplot [74] was employed to draw the chloroplast genome structure of *Eriocaulon.* All of the new sequenced and annotated complete chloroplast genomes were deposited in GenBank and the accession numbers were shown in Table S1. Geneious Prime was used to extract the protein-coding genes of *Eriocaulon* chloroplast genomes. Relative synonymous codon usage (RSCU) indicated the ratio of the observed frequency of a particular codon to the expected frequency of that codon. The codon frequency and RSCU were calculated using MEGA X and codon frequency distribution was illustrated using TBtools [75] with the form of a heatmap.

### Chloroplast genome sequence divergence analysis

To visualize the sequence divergence among the *Eriocaulon* species, the mVISTA program was used to compare the 18 *Eriocaulon* species’ chloroplast genomes. The annotation of *Eriocaulon alpestre* (OK539714) was used as a reference. To identify the mutation hotspot regions and quantize the sequence divergence, we aligned the 20 chloroplast genomes with MAFFT v7.0. Variable and parsimony-informative sites, and nucleotide diversity (π) in the aligned sequences were used to evaluate sequence divergence. Variable and parsimony-informative sites were calculated with MEGA X [76]. The π value was calculated with the software DnaSP v6 [77] using the sliding window method. The window length was set to 800 bp with a 100-bp step size.

### Simple sequence repeats and repeat structure analysis

Four types of repeat sequences, forward, palindromic, reverse, and complement repeats, were identified by the REPuter online program [78] with the parameters of a repeat size of ≥ 30 bp and a Hamming distance of 3. SSRs were identified using the PERL script microsatellite identification (MISA) software [79], with the threshold number of repeats set as ≥ 10 repeat units for mononucleotides, ≥ 5 for dinucleotides, ≥ 4 for trinucleotides, and ≥ 3 for tetranucleotides, pentanucleotides, and hexanucleotides.

Molecular evolution of the chloroplast genome of *Eriocaulon*. We used the ratio (ω) of non-synonymous (dN) to synonymous (dS) substitutions to analyze the role of natural selection in driving the molecular evolution of the *Eriocaulon* chloroplast genome. The ω value is an indicator of natural selection of the protein-coding genes. The values ω > 1, ω = 1, and ω < 1 indicate positive, neutral, and negative selection, respectively. All the protein-coding genes were aligned with the MAFFT and deleted the stop codon. The dN, dS and ω values were calculated using the MEGA X [76]. We analyzed all the 79 protein-coding genes and the gene groups with some function, such as *atp*, *psa*, *pet*, and *rpo.*

### Phylogenetic analyses

Both maximum likelihood (ML) and Bayesian inference (BI) methods were performed to infer the phylogeny relationships of *Eriocaulon*. We used two datasets to infer phylogenic relationships: the complete chloroplast genome sequences and the 83-genes (including 79 protein-coding genes and four rRNA genes) of the 32 samples, with *Paepalanthus alpinus* as the outgroup. The nucleotide sequences of the 79 common protein-coding genes were extracted from each chloroplast genome, aligned, and concatenated.

Best-fitting models of nucleotide substitution were selected using ModelFinder [80]. ML analyses were performed in RAxML-NG [81] with 500 bootstrap replicates (BS). The BI analysis was performed in Mrbayes v3.2 [82] with two independent Markov chain Monte Carlo chains. Each chain began with a random tree with 2,000,000 generations. The first 25% of the sampled trees were discarded as burn-in, and the Bayesian posterior probabilities (PP) were calculated using the remaining trees.

### Phylogenetic analyses using the chloroplast gene sequences from GenBank

Five chloroplast genes of *rbcL*, *rpoB*, *matK*, *rpoC1*, and *trnL-F* of *Eriocaulon* were downloaded from GenBank database. All the genes were aligned using MAFFT, and concatenated by the information of specimen voucher in order to ensure these sequences from the same individual using PhyloSuite v1.2.2 [83]. The ML tree was reconstructed using the IQ-TREE v2 and the supported values were assessed used the ultrafast bootstrap approximation (UFBoot) methods [84].

### Fossil priors and divergence time estimate

Divergence time was estimated using BEAST v2.5.1 [85] with two priors based on the concatenated 83-gene dataset and the five chloroplast gene dataset (keep one sample of each species). Following Larridon et al. [4], two priors were used: (i) the crown age of Eriocaulaceae was 56 Ma (the root of the tree); (ii) the crown age of *Eriocaulon* was 21.66 Ma.

Uncorrelated log-normal distribution relaxed molecular clock models were selected to account for rate variability among clades. The nucleotide substitution model and the prior tree model were set to GTR and Yule models, respectively. Both priors were set under the normal distribution. The MCMC run had a chain length of 500,000,000 generations with sampling every 10,000 generations. Tracer 1.6 [86] was used to evaluate convergence and ensure a sufficient and effective sample size for all parameters surpassing 200. The maximum clade credibility tree was produced using TreeAnnotator v2.4 after discarding the first 10% of the generations.

## Supplementary Information

**Additional file 1:** **Figure S1.** The RSCU values of the coding genes in the *Eriocaulon* chloroplast genome.

**Additional file 2:** **Figure S2.** mVISTA-based sequence identity plot of 18 *Eriocaulon* species, using *E. alpestre* as a reference.

**Additional file 3:** **Figure S3.** Phylogenetic trees of Eriocaulon. a. The whole chloroplast genome dataset. b. The 83-genes dataset. The number above the lines indicates the ML bootstrap values (BS) and BI posterior probability (PP). BS=100 and PP=1.0 are not shown.

**Additional file 4: Figure S4.** Divergence times of *Eriocaulon* using the five chloroplast genes.

**Additional file 5:** **Table S1.** Sampling information for the *Eriocaulon* samples in this study.

**Additional file 6:** **Table S2.** The dN, dS, and ω values of 79 protein-coding genes. 

**Additional file 7:** **Table S3.** The GenBank information of the chloroplast genes used in inferring the *Eriocaulon* phylogeny.

## Data Availability

New sequenced and other published chloroplast genome sequences can be found in GenBank (https://www.ncbi.nlm.nih.gov/genbank/), and the accession numbers showed in Table S1.
