# Supplementary figures and images for: Insights into the phylogeny and chloroplast genome evolution of Eriocaulon (Eriocaulaceae)
Source: BMC Plant Biol. 2023 Jan 14;23:32. doi: 10.1186/s12870-023-04034-z (PMC9840334; doi:10.1186/s12870-023-04034-z)

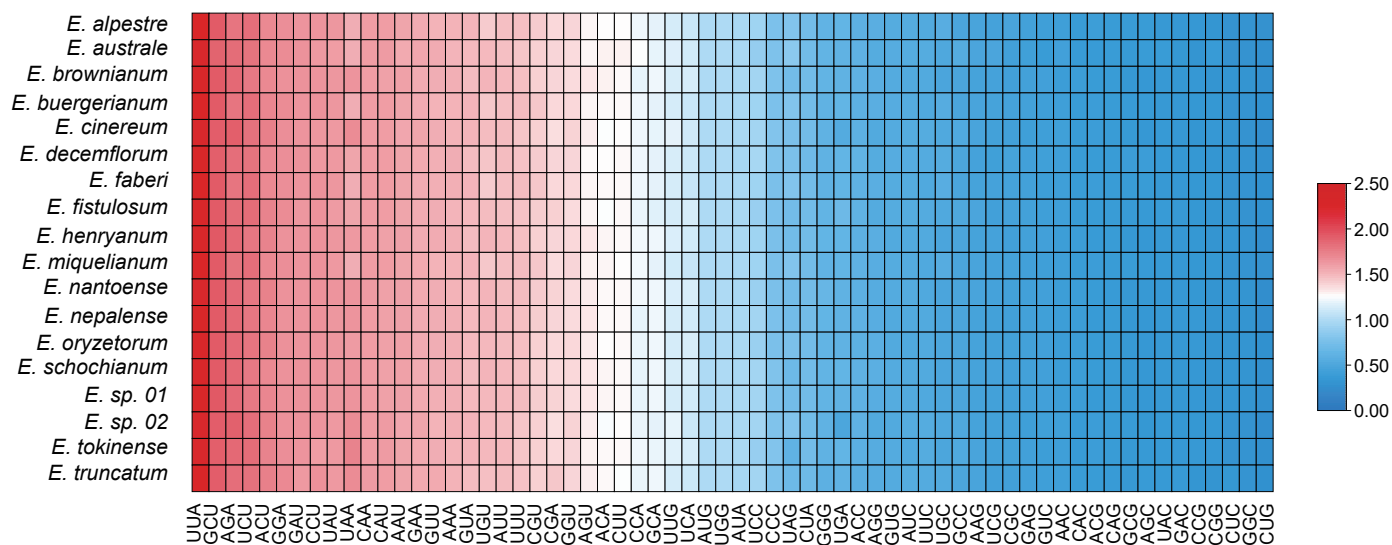

**Figure S1.** The RSCU values of the coding genes in the *Eriocaulon* chloroplast genome.

Supplement: Supplementary file 1 — Additional file 1: Figure S1. The RSCU values of the coding genes in the Eriocaulon chloroplast genome. [file 12870_2023_4034_MOESM1_ESM.pdf]

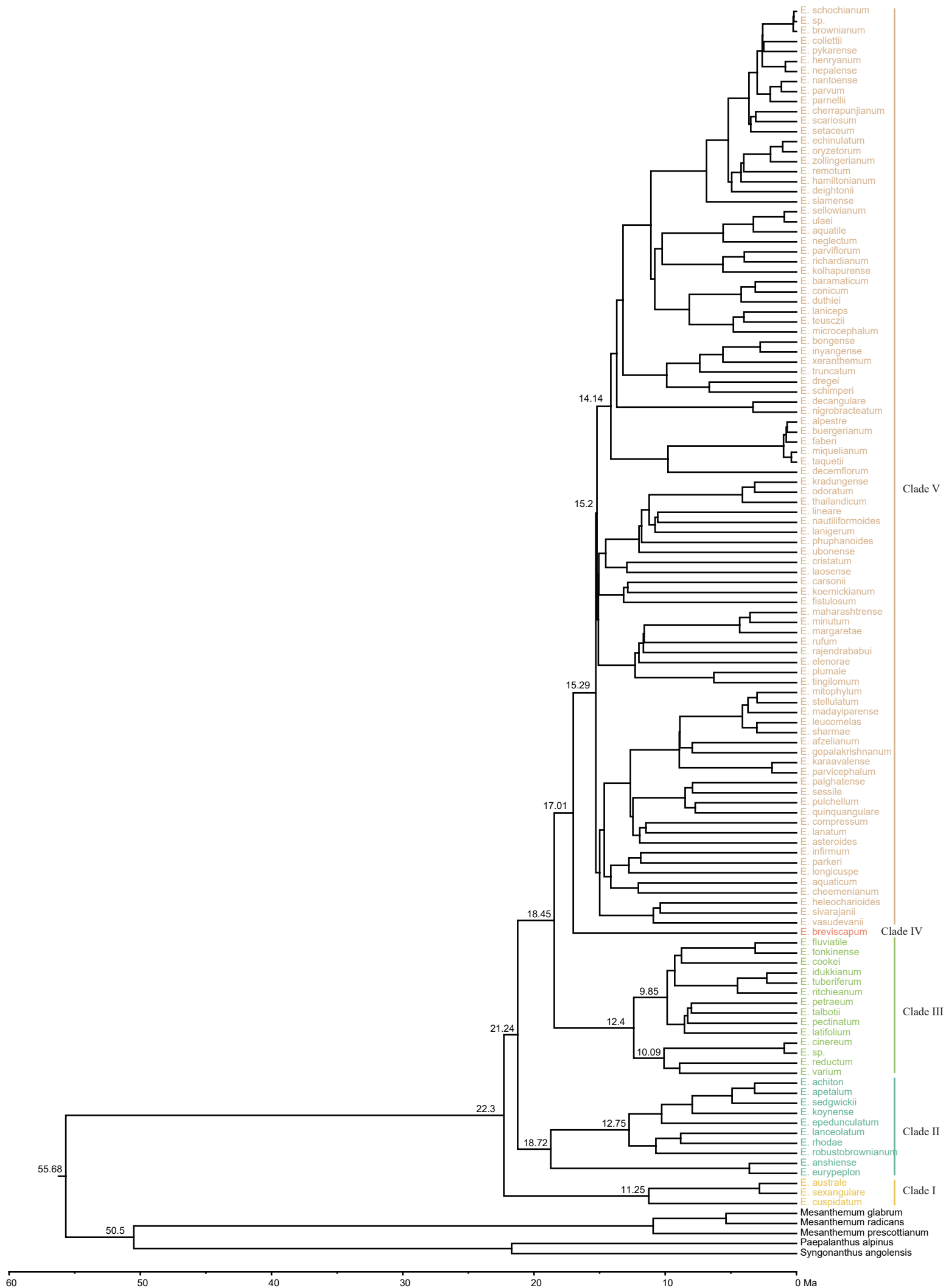

**Figure S4.** Divergence times of *Eriocaulon* using the five chloroplast genes.

Supplement: Supplementary file 4 — Additional file 4: Figure S4. Divergence times of Eriocaulon using the five chloroplast genes. [file 12870_2023_4034_MOESM4_ESM.pdf]
