# Supplementary material for: Insights into the phylogeny and chloroplast genome evolution of Eriocaulon (Eriocaulaceae)
Source: BMC Plant Biol. 2023 Jan 14;23:32. doi: 10.1186/s12870-023-04034-z (PMC9840334; doi:10.1186/s12870-023-04034-z)
Supplement: Supplementary file 2 — Additional file 2: Figure S2. mVISTA-based sequence identity plot of 18 Eriocaulon species, using E. alpestre as a reference. [file 12870_2023_4034_MOESM2_ESM.pdf]

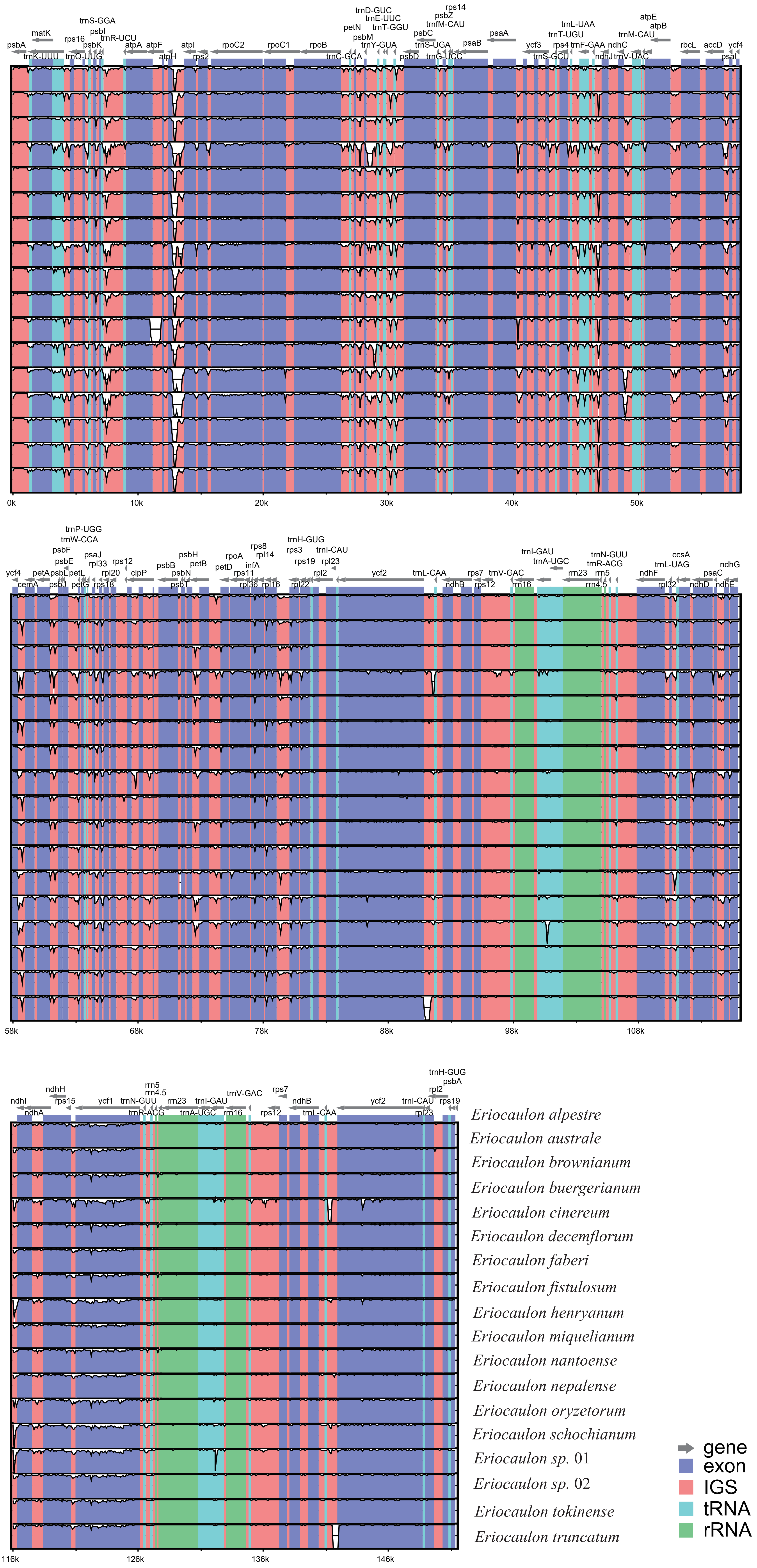

Figure S2. mVISTA-based sequence identity plot of 18 Eriocaulon species, using *E. alpestre* as a reference.
