# Supplementary material for: Insights into the phylogeny and chloroplast genome evolution of Eriocaulon (Eriocaulaceae)
Source: BMC Plant Biol. 2023 Jan 14;23:32. doi: 10.1186/s12870-023-04034-z (PMC9840334; doi:10.1186/s12870-023-04034-z)
Supplement: Supplementary file 3 — Additional file 3: Figure S3. Phylogenetic trees of Eriocaulon. a. The whole chloroplast genome dataset. b. The 83-genes dataset. The number above the lines indicates the ML bootstrap values (BS) and BI posterior probability (PP). BS=100 and PP=1.0 are not shown. [file 12870_2023_4034_MOESM3_ESM.pdf]

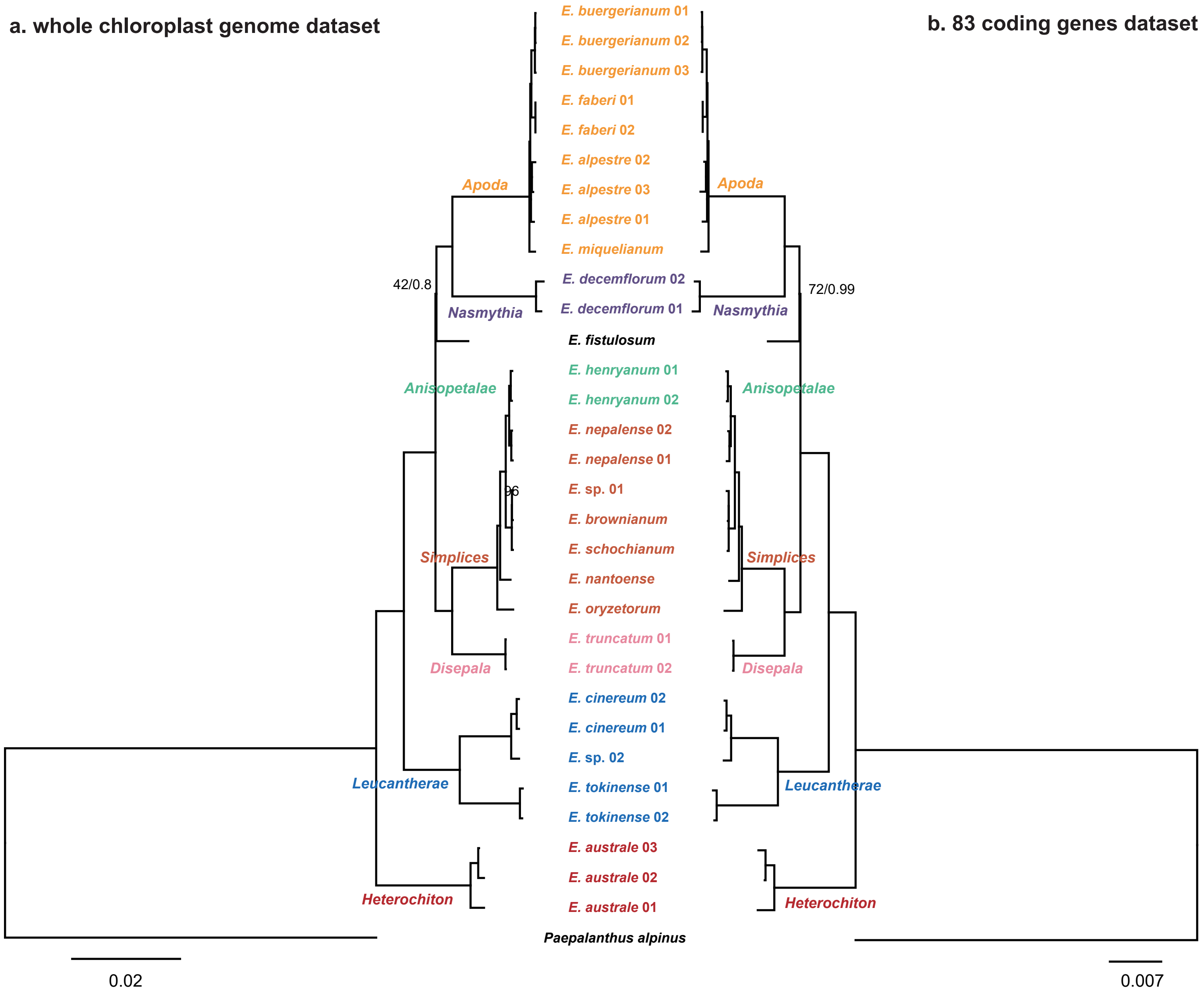

Figure S3. Phylogenetic trees of Eriocaulon. a. The whole chloroplast genome dataset. b. The 83-genes dataset. The number above the lines indicates the ML bootstrap values (BS) and BI posterior probability (PP). BS=100 and PP=1.0 are not shown.
